# Calcium accumulation or iron deposition: Delving into the temporal sequence of amyotrophic lateral sclerosis pathophysiology in the primary motor cortex

**DOI:** 10.1002/ibra.12168

**Published:** 2024-06-28

**Authors:** Sadegh Ghaderi, Sana Mohammadi, Farzad Fatehi

**Affiliations:** ^1^ Department of Neuroscience and Addiction Studies, School of Advanced Technologies in Medicine Tehran University of Medical Sciences Tehran Iran; ^2^ Neuromuscular Research Center, Department of Neurology, Shariati Hospital Tehran University of Medical Sciences Tehran Iran; ^3^ Department of Neurology University Hospitals of Leicester NHS Trust Leicester UK

**Keywords:** ALS, calcium, iron, primary motor cortex, QSM

## Abstract

Amyotrophic lateral sclerosis (ALS) causes progressive motor neuron degeneration, but an in vivo understanding of its early pathology remains limited. A recent study used topographic layer imaging to investigate iron and calcium accumulation in the primary motor cortex (M1) of patients with ALS compared with controls. Despite the preserved cortical thickness, ALS patients showed increased iron in layer 6 and calcium accumulation in layer 5a and the superficial layer. Calcium accumulation was particularly prominent in the low‐myelin borders, potentially preceding the demyelination. This study reveals a novel in vivo pathology in ALS, suggesting that calcium dysregulation may precede iron accumulation and contribute to early M1 cell degeneration. Further investigation using quantitative susceptibility mapping and complementary techniques, such as diffusion kurtosis imaging, along with ultrahigh‐field magnetic resonance imaging, into the role of calcium and early intervention strategies is warranted.

## Graphical abstract

Amyotrophic lateral sclerosis (ALS) causes motor and nonmotor neuron degeneration, and the understanding of its early pathology is limited. A recent study used imaging to investigate iron and calcium accumulation in the primary motor cortex (M1) of ALS patients compared with controls. Despite preserved cortical thickness, ALS patients showed increased iron in layer 6 and calcium accumulation in layer 5a and the superficial layer. Calcium accumulation was prominent in low‐myelin borders, potentially preceding demyelination. This study reveals a novel pathology in ALS, suggesting that calcium dysregulation may precede iron accumulation and contribute to early M1 cell degeneration. Further research using quantitative susceptibility mapping and additional methods such as diffusion kurtosis imaging, in combination with ultrahigh‐field magnetic resonance imaging, is necessary to explore the impact of calcium and potential early intervention approaches.

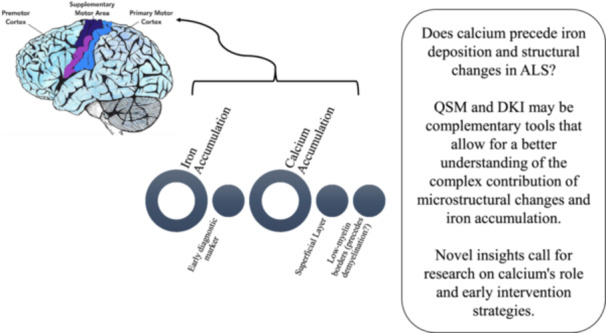

Northall et al.^1^ conducted interesting research on multimodal layer modeling revealing in vivo pathology in amyotrophic lateral sclerosis (ALS). They used topographic layer imaging to study the in vivo pathology of patients with ALS, which allowed them to test averaged quantitative T1 and quantitative susceptibility mapping (QSM) values across cortical fields (lower limb [LL], upper limb [UL], and face [F]) and layers (layer 6 [L6], layer 5a [L5a], layer 5b [L5b], and superficial layer [Ls]) of the primary motor cortex (M1). In this letter, we discuss the novel findings presented by Northall et al.^1^ regarding the in vivo pathology of ALS and their potential implications for understanding the temporal sequence of disease progression.

M1 is a crucial region that is responsible for planning and executing voluntary movements.^1^ Located in the precentral gyrus, just anterior to the central sulcus, it is organized into six distinct layers: molecular, outer granular, outer pyramidal, granular (not well‐developed), inner pyramidal, and multiform.^1^ It is acknowledged that degeneration of specific motor layers in the brain is a characteristic of ALS.^1^, ^2^

Depopulation of Betz cells in the M1 and axonal loss in the descending motor pathway associated with myelin pallor and gliosis of the corticospinal tract are hallmarks of upper motor neuron (UMN) involvement in ALS.^1^ Previous postmortem studies have also confirmed abnormalities in M1 in ALS, such as depopulation of Betz cells in the cortical L5b and Betz cell loss.^3^, ^4^ Before the Northall et al. study, previous magnetic resonance imaging (MRI) studies based on susceptibility‐based techniques reported changes in M1 due to increased iron accumulation, including increased QSM value, hypointensity in susceptibility‐weighted imaging (SWI), and T2* shortening.^2^, ^5^, ^6^ These studies suggest that iron accumulation in the M1 of ALS patients is caused by iron accumulation within ferritin‐laden microglial cells. Thus, previous studies have confirmed that Betz cells are affected by ALS. This degeneration is linked to the accumulation of iron,^2^ microscopic protein aggregation,^7^ and calcium dysregulation in the M1.^8^

Notably, MRI postprocessing techniques such as QSM, used in the study by Northall et al.,^1^ offer superior sensitivity to detect subtle changes in tissue composition, such as iron accumulation, compared with conventional MRI (Figure 1). Furthermore, advancements such as ultrahigh‐field MRI promise even greater resolution, potentially enabling the visualization of finer pathological changes within the cortical layers. The integration of multimodal neuroimaging techniques holds promise for a more comprehensive understanding of ALS pathology. For instance, diffusion kurtosis imaging (DKI) can assess the white matter and gray matter microstructure using non‐Gaussian approaches without the importance of spatial distributions, potentially revealing disruptions in interregional communication that complement the findings of QSM.^9^, ^10^ By combining these advanced MRI techniques, researchers can gain a more holistic view of the pathological cascade in ALS, paving the way for earlier diagnosis and development of targeted therapies.

**Figure 1:**
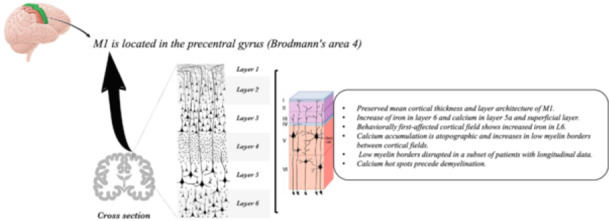
Summary of Northall et al.'s^1^ findings on cortical thickness and layer architecture in patients with amyotrophic lateral sclerosis. M1, primary motor cortex. [Color figure can be viewed at wileyonlinelibrary.com]

While previous research has confirmed significant Betz cell degeneration in ALS within L5 of the M1, multimodal layer modeling by Northall et al.^1^ presents intriguing new findings. Despite the preserved cortical thickness and architecture, this study revealed elevated iron levels in L6 and calcium accumulation in both L5a (containing Betz cells) and the superficial layer of patients with ALS. The behaviorally first‐affected cortical field showed increased iron in L6, whereas calcium accumulation was atopographic and increased in the low‐myelin borders between the cortical fields. The low myelin borders were disrupted in a subset of patients with longitudinal data. These findings suggest a potential shift in our understanding of the ALS pathology. The observed calcium accumulation before iron deposition and before evident structural changes raises the possibility that calcium dysregulation and its subsequent accumulation might precede and contribute to the disruption and destruction of M1 L5 cells, which are responsible for motor control. Furthermore, this study identified calcium accumulation in the low‐myelin borders, potentially hinting at early disruptions in interregional communication before overt demyelination. These novel insights highlight the need for further rigorous investigation of the precise role of calcium dysregulation and its potential contribution to the early stages of M1 cell degeneration in ALS. Overall, this study revealed that changes in tissue microstructure precede cortical atrophy, iron accumulation is a diagnostic marker, and calcium accumulation may precede demyelination and cell loss. Elucidating the temporal and causal relationships between calcium accumulation, iron deposition, and subsequent cellular damage could pave the way for novel therapeutic strategies targeting these early pathological processes in ALS.

In conclusion, the findings of Northall et al.^1^ suggest a potential shift in our understanding of the ALS pathology. The observed calcium accumulation preceding iron deposition challenges the traditional view and highlights the need for further investigation of the role of calcium dysregulation in the early stages of the disease. Furthermore, the identification of calcium accumulation in low‐myelin borders raises intriguing questions about its potential contribution to early disruptions in interregional communication before overt demyelination is observed. Elucidation of the temporal and causal relationships among calcium accumulation, iron deposition, and subsequent cellular damage may pave the way for novel therapeutic strategies targeting these early pathological processes in ALS.

## AUTHOR CONTRIBUTIONS

Sadegh Ghaderi and Sana Mohammadi contributed to the conceptualization, methodology/study design, software, data curation, original draft preparation, visualization, and review and editing of the manuscript; Farzad Fatehi contributed to the conceptualization, methodology, supervision, review, and editing of the manuscript.

## CONFLICT OF INTEREST STATEMENT

The authors declare no conflict of interest.

## ETHICS STATEMENT

Not applicable.

## Data Availability

Not applicable as no new data are generated in this letter.
